# Assessment of red blood cell deformability in type 2 diabetes mellitus and diabetic retinopathy by dual optical tweezers stretching technique

**DOI:** 10.1038/srep15873

**Published:** 2016-03-15

**Authors:** Rupesh Agrawal, Thomas Smart, João Nobre-Cardoso, Christopher Richards, Rhythm Bhatnagar, Adnan Tufail, David Shima, Phil H. Jones, Carlos Pavesio

**Affiliations:** 1 Moorfields Eye Hospital NHS Foundation Trust, London, UK; 2 Department of Mechanical Engineering, University College London, London, UK; 3 Institute of Ophthalmology, University College London, London, UK; 4 National Healthcare Group Eye Institute, Tan Tock Seng Hospital, Singapore; 5 Department of Physics and Astronomy, University College London, London, UK

## Abstract

A pilot cross sectional study was conducted to investigate the role of red blood cells (RBC) deformability in type 2 diabetes mellitus (T2DM) without and with diabetic retinopathy (DR) using a dual optical tweezers stretching technique. A dual optical tweezers was made by splitting and recombining a single Nd:YAG laser beam. RBCs were trapped directly (i.e., without microbead handles) in the dual optical tweezers where they were observed to adopt a “side-on” orientation. RBC initial and final lengths after stretching were measured by digital video microscopy, and a Deformability index (DI) calculated. Blood from 8 healthy controls, 5 T2DM and 7 DR patients with respective mean age of 52.4yrs, 51.6 yrs and 52 yrs was analysed. Initial average length of RBCs for control group was 8.45 ± 0.25 μm, 8.68 ± 0.49 μm for DM RBCs and 8.82 ± 0.32 μm for DR RBCs (p < 0.001). The DI for control group was 0.0698 ± 0.0224, and that for DM RBCs was 0.0645 ± 0.03 and 0.0635 ± 0.028 (p < 0.001) for DR group. DI was inversely related to basal length of RBCs (p = 0.02). DI of RBC from DM and DR patients was significantly lower in comparison with normal healthy controls. A dual optical tweezers method can hence be reliably used to assess RBC deformability.

Diabetic retinopathy (DR) is an important microvascular complication of diabetes which leads to blindness in the working age population (20–64 years old) in developed countries^1^. The pathogenesis of DR is multifactorial but is primarily caused by the metabolic effects of chronic hyperglycemia, which result in vascular changes resulting in retinal ischemia^1^,^2^. More advanced retinal disease, including proliferative vascular changes and neovascularization in the setting of retinal ischemia, may be mediated by other mechanisms such as the action of vasoactive substances released during the inflammatory process. There is compelling evidence suggesting a significant role of inflammation and hyperglycemia in development of DR^1^,^3^. There are some reports showing decreased deformability of polymorphonuclear leukocytes or white blood cells (WBC) in type 2 diabetes mellitus (T2DM)^4^. However, inflammation and hyperglycemia have been shown to be associated with the ultra-structural and functional changes in erythrocytes or red blood cells (RBCs)^5^. There is decrease in the capillary diameter in diabetic microangiopathy because of the thickening of the basal membrane and accumulation of the several metabolites^1^. Appropriate compliance or flexibility of RBCs is required to prevent disturbance to the blood flow in the microcirculation and to prevent occlusion and consequently hypoxia^6^.

With advances in the knowledge about erythrocyte dynamics and its implications in numerous hemorheological disorders, there is a sudden surge in interest in looking at different aspects of RBC properties in vascular ischaemic disorders. With significant vascular inflammatory and occlusive events happening in the eye either in isolation or as a part of systemic disease, it will be interesting to revisit some of the basic properties of RBCs as a contributory, if not root cause, for some of this ocular vascular occlusive disorders. Erythrocyte deformability or the ability of the red blood cell (RBCs) to change its shape under applied stress is an important function of the erythrocyte. In the microcirculation, the capillary diameter ranges between 2 to 10 μm, whereas the average diameter of RBCs is 7.5 μm. Deformability allows RBCs to pass through the smaller vessels in the circulation while maintaining its normal function^7^,^8^,^9^,^10^,^11^.

There are numerous factors that can affect RBC deformability^5^,^9^,^12^,^13^,^14^,^15^ and also there are many different ways to assess RBC deformability^5^,^10^,^16^,^17^,^18^,^19^. The common factor analysed using the available techniques is ability of RBC to alter their shape in response to the applied shear stress imposed either by the momentum of the viscous fluid flow or by a stationery barrier^20^.

Optical tweezers are a powerful technique for the manipulation of matter on the micron and sub-micron scale^21^, and have been applied to a range of inorganic materials from nanotubes to microbubbles^22^,^23^. As a method of non-contact trapping they are advantageous for biological material including viruses and bacteria and single cells^21^,^22^,^23^,^24^,^25^. Optical forces have been used to assess the deformability of red blood cells using conventional optical tweezers^26^,^27^,^28^ and also the “optical stretcher” method^29^. Multiple trap optical tweezers set up have been proposed in the past to measure the elastic properties of the cell and to determine the elastic moduli of the cell if the cell is osmotically swollen to adopt a spherical shape^28^,^29^. Optical force has either been applied using microbeads attached to the cells as handles^30^, or by direct application of laser beam to the cells^31^. Liao *et al*. have previously studied the transverse extent of stretch of the biconcave RBCs using direct application of a single trapping beam rapidly jumping between two positions and the cell’s elongation was reported as a measure of the extent of deformability^31^. RBC deformability has been associated with the diabetic kidney disease but its relationship to ocular disease is less clear^15^. There have been limited attempts to correlate the biochemical and other haematological parameters affecting the RBC deformability in diabetes^15^,^17^,^32^ and there are no published studies looking at the initial length and morphology of the RBC as a determinant factor for deformability of the cell.

The primary objective of the study was to investigate the RBC deformability in patients with retinal vascular disorders such as DR in comparison to RBC deformability of patients with T2DM and healthy normal controls using a dual optical tweezers stretching method. A secondary objective was to analyse the factors affecting RBC deformability in normal healthy controls and diabetes patients.

## Methodology

All the research was performed as per declaration of tenets of Helsinki. Ethics board approval was obtained (14/WM/1038). The study was approved by Coventry and Warwick NRES Committee, West Midlands (REC ref: 14/WM/1038). Study was conducted between August 2014 and July 2015. Five patients without DR and seven patients with mild non proliferative DR in Type 2 Diabetes Mellitus (T2DM) were consented for the study. Eight age and gender matched control subjects were recruited for the study. Informed consent was obtained from all the participants. Details about the demographics and personal history were recorded on a pre-coded data information sheet.

### Collection of the blood

Blood samples were collected using a vacutainer (BD vacutainer™ Blood collection needles, 21G × 1inch; sleeve wall: thin ) from the antecubital vein without interruption of the venous flow. All the blood samples were collected at noon (just before the lunch) with subjects in a sitting position to avoid the potential confounders secondary to time and posture. 20 ml of the blood was collected for full blood count [haemoglobin (Hg), hematocrit (Hct), RBC count, mean corpuscular volume (MCV), mean corpuscular haemoglobin (MCH), mean corpuscular haemoglobin concentration (MCHC), red blood cell distribution width (RDW), platelet concentration (PC), mean platelet volume (MPV), white blood cell count (WBC), Neutrophils (PMNs), Lymphocytes, Monocytes, Eosinophils, Basophils, erythrocyte sedimentation rate (ESR), serum electrolytes [sodium (Na), potassium (K), chloride (Cl), bicarbonates], renal function test [blood urea, serum creatinine, glomerular filtration rate (GFR), serum bilirubin], liver function test (alkaline phosphtase (ALP), alanine aminotransferase (ALT), aspartate aminotransaminase,(AST) ), total proteins, albumin, globulin), C-reactive protein (CRP), random blood glucose (RBG), glycosylated haemoglobin (HbA1C), lipid panel [triglycerides (TG), total cholesterol, high density lipoproteins (HDL), low density lipoproteins (LDL), serum angiotensin converting enzyme (ACE) and fibrinogen. 4 ml of the blood was collected in a spray-coated K2-EDTA (plastic) BD^®^ vacutainer tubes with BD Hemogard™ closure for assessment of RBC deformability.

### Transport of blood sample

In addition to routine laboratory test, 4 ml of the blood were sent in EDTA tube to the department of physics and astronomy at University College London in a labelled biobox. The temperature of the biobox was kept at around 4 °C and the total transfer time for blood from the phlebotomist room to the research laboratory was 30–45 mins.

### Preparation of the slide

At the research laboratory at Department of Physics and Astronomy, under sterile conditions the well slide was prepared (RB/RA/JC/TS/CR) by diluting the RBC sample (6 × 10^–^^4^ ) with phosphate buffer saline (PBS) buffer and with 1% Bovine Serum Albumin to prevent formation of aggregates and also to prevent cells from adhering to the slides. (Appendix 1).

### Optical tweezers

**Figure 1:**
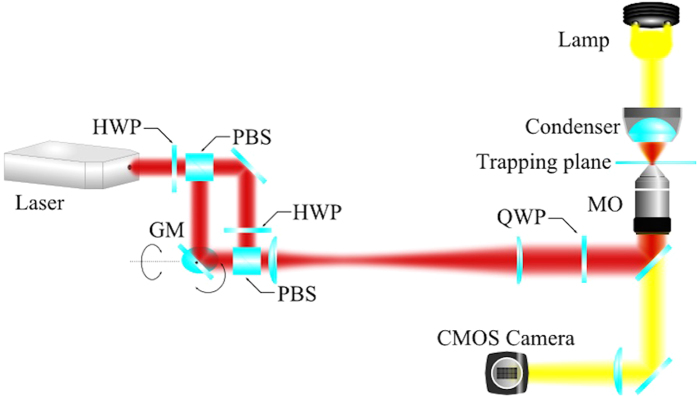
Schematic diagram showing set up of dual-beam optical tweezer: Experimental set up. (HWP = half-wave plate, QWP = quarter-wave plate, PBS = polarising beam splitter, MO = microscope objective, CMOS = Complementary Metal Oxide Silicon).

A dual optical tweezers (two trapping beams) was set up to assess the red blood cell deformability (PJ). The dual optical tweezers set up was made by splitting and recombining a single Nd: YAG laser beam. One of the beams was sent via orthogonally mounted galvanometer scanning mirrors located in a plane conjugate to the objective back aperture so that the position of the optical trap it formed can be controlled by computer in two dimensions. The two beams were expanded to slightly overfill the back aperture of the objective lens and injected into the fluorescence port of an inverted microscope.The beams were focused to diffraction-limited spots by a high numerical aperture (NA = 1.3, oil immersion) ×100 objective which also served to image the cells onto a CMOS camera (Fig. 1).

### Microbead handles

Previous optical tweezers experiments have applied stretching force either directly or via attached microbead handles. Since direct trapping is simpler and still capable of producing a measurable deformation of the RBC^31^ we decided to proceed with this technique. Before acquiring data we verified that for the optical power used there was no change in the properties of the cell that occurred for duration of laser illumination several times longer than the length of a typical stretching experiment.

**Figure 2:**
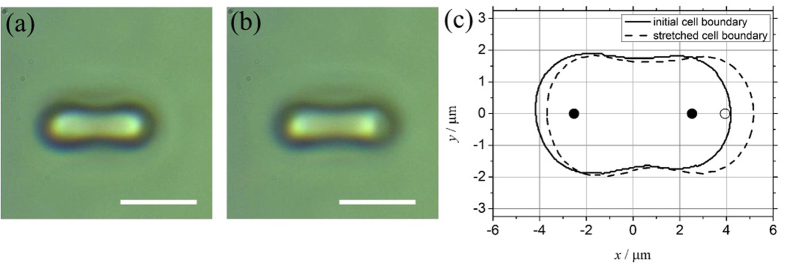
Side on images of RBC under dual beam optical tweezer set up. (**2a**) Unstretched RBC: Side on view of a unstretched RBC (scale bar: 5 um). (**2b**) Stretched RBC: Side on view of a Stretched RBC (scale bar: 5 um). (**2c**) RBC boundaries demarcated – continuous edges representing unstretched RBC, broken edges representing stretched RBC, black dots indicates laser spots at the start and white dots indicate the laser spot position at the time of maximum stretch.

RBCs were trapped directly in the dual optical tweezers where they adopt an orientation as seen in images from the experiments, i.e. they are observed “side-on” (Fig. 2A). The initial separation of the optical traps was 5.06 μm. The cells were stretched by increasing the trap separation (moving one beam only) at a rate of 0.47 μm/s for 3 seconds, to 6.47 μm (Fig. 2B). The cell was released by quickly jumping one of the traps to a large distance away (a separation of 18.64 uμm) where it no longer affected the RBC, and was held there for 1 second while the cell relaxed to its initial length. The cell was then recaptured by jumping the trap to a separation of 2.08 μm, and then brought back to its initial state by ramping back to its initial position over 1 second, i.e. 2.92 μm/s.

An experimental sequence consists of 5–10 of the above cycles per cell (see supplementary Video 1). Each cell was subjected to 3 × 5–10 cycles in a time of less than 5 minutes and for each sample we attempted to assess 10 random cells. Measurement of the initial transverse length (Io) in μm and final stretched length of the RBC (Imax) in μm was done via digital video microscopy. A Matlab™ code was used to extract the boundary of the cell from each frame of the video and the extreme of the bounding curve used as a measure of the length of the cell. The measured cell boundary for the initial and fully stretched cell along with the relative position of the trapping laser spots is shown in Fig. 2C (TS**/**CR).

### Deformability index (DI)

There are different formulae proposed in the literature as an indicator of RBC deformability. In our current study, we used a relatively simple formula as a measure of RBC deformability:
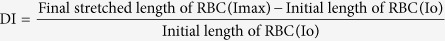


### Statistical tests

Qualitative variables were expressed as percentages. Quantitative variables were expressed as mean values ± standard deviation (SD) if they followed a normal distribution or as median values (range) if not. Correlation for all the blood parameters between control group and study group was performed using either Fisher’s exact test or Χ^2^ test. Wilcoxon sign rank test and ttest, was used to study the difference in means for Io, Imax, DI between study group and control group. Univariate regression analysis was performed with the DI as the dependent variable. Variables showing significant association with DI on univariate analysis were included in the multivariate analysis model. Results were considered statistically significant when p < 0.05. The data was analysed using Stata ⁄ SE, version 13.0 (Stata Corp, College Station, TX, USA).

## Results

For this pilot study, a total of 5 patients with T2DM without DR (DM – study group 1), (mean age-51.6yrs, 3(60%) males), 7 patients with T2DM and associated diabetic retinopathy (DR - study group 2) (mean age: 54.71yrs, 4(50%) males) and 8 control subjects (control group) (mean Age: 52.37yrs, 5(71.43%) male) were consented and recruited.

The routine blood test, liver function test, cholesterol profile, renal function test and coagulation profile were compared between the control group and DR group and are presented in the tabular format (Table 1). Blood from patients with T2DM (study group 1) was matched for HbA1C and random blood glucose and there was no statistically significant difference in the two study groups for any of the biochemical or hematological parameters in the blood. Besides RBG and HbA1 which were significantly elevated in the study group (p < 0.001), ALP (p < 0.05), total protein (p < 0.05), globulin (p < 0.001) were also found to be elevated in the DR subjects. MCV (p = 0.01) and MCH (p = 0.02) were found to be reduced in patients with DR.

### Dual optical tweezers

Video of the experiments was recorded at rates of up to 80 frames per second. There was slippage of the cell from the trap if same cell was trapped for longer time for more than 2 minutes. Overall cells looked healthy in optimal concentration and there were less than 5% echinocytes per slide and hence not affecting the assessment of cells under two directional dual-beam optical tweezers. Other observations noted were intermittent rotation of the cells while being trapped within the laser.

Io for control group was 8.45 μm (±0.25, Range: 8.03–9.25), 8.68 (±0.49, Range: 7.33–10.27) for DM RBCs and 8.82 μm (±0.32, Range: 8.03–9.62) for DR RBCs (p < 0.001, Wilcoxon sign rank test) (Table 2). Imax using dual-beams optical tweezers was 9.04 μm (±0.17, Range: 8.55–9.65) for control group, 9.23 μm (±0.54, Range: 7.82–10.56) for DM group and 9.39μm (±0.26, 95CI: 8.70–10.03) for DR group (p < 0.001, Wilcoxon sign rank test) (Table 2). The difference between Imax and Io was computed and mean for both control and study group (p < 0.001, Wilcoxon sign rank test) is presented in Table 2. The DI for control group was 0.0698 (±0.0224, Range: 0.016–0.131), for DM RBCs this was 0.0645(±0.038, Range: 0.015–0.188) and that for DR RBCs was 0.0635 (±0.028, Range: 0.014–0.129) (p < 0.001, Wilcoxon sign rank test) (Table 2).

**Figure 3:**
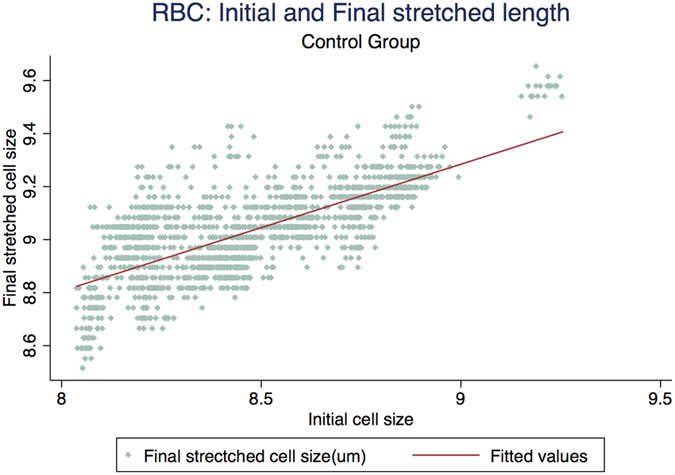
Scatter plot representing the linear relationship between initial unstretched length of RBCs with final stretched length of RBCs in the control group.

**Figure 4:**
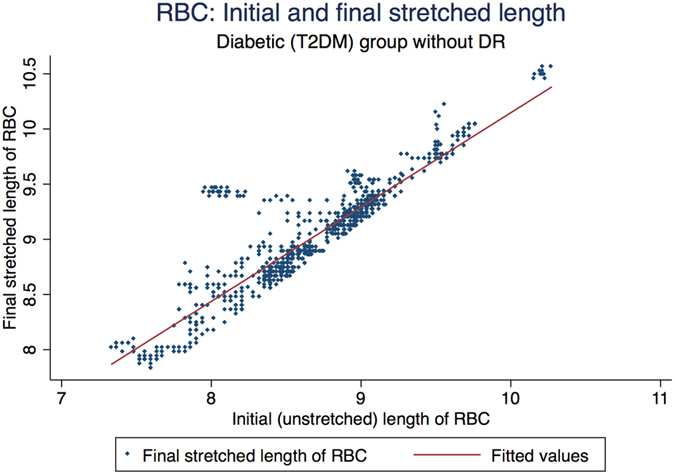
Scatter plot representing the linear relationship between initial unstretched length of RBC with final stretched length of RBCs in the Type 2 diabetes mellitus group without diabetic retinopathy.

**Figure 5:**
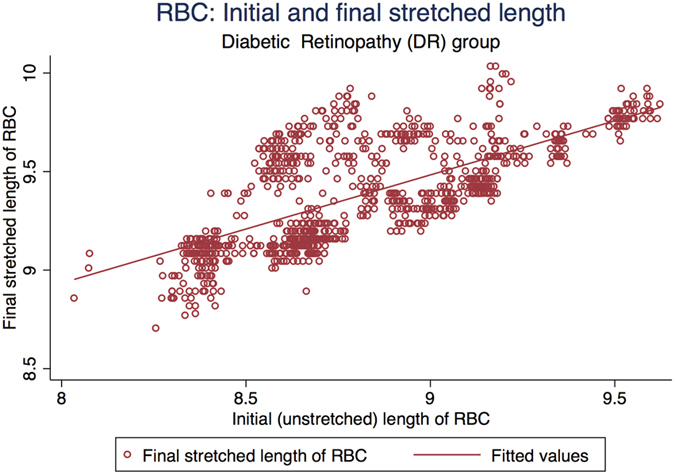
Scatter plot representing the linear relationship between initial unstretched length of RBC with final stretched length of RBCs in the diabetic retinopathy group.

**Figure 6:**
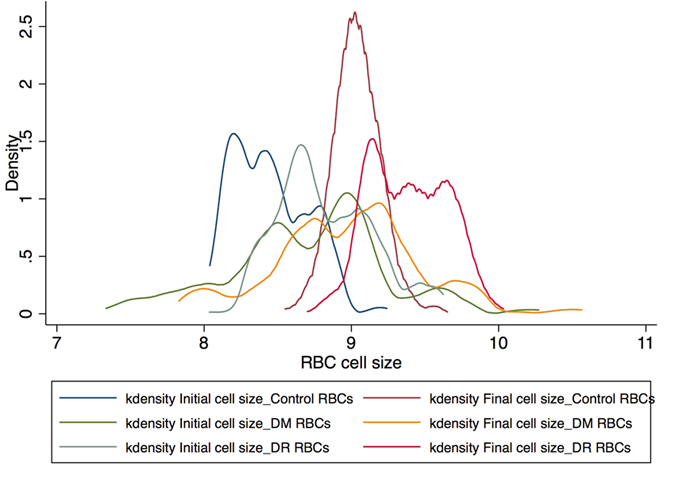
Kernel Density Plots for Initial and Stretched cell sizes for control and diabetic retinopathy group.

**Figure 7:**
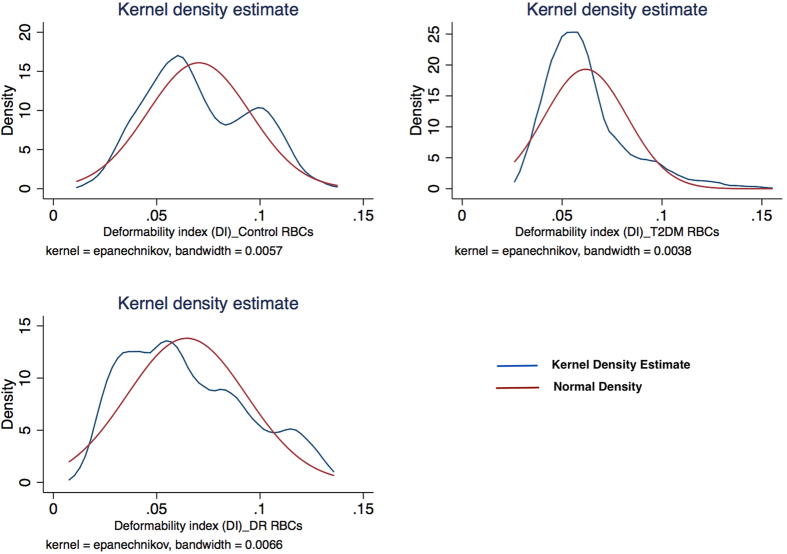
Kernel Density estimate plots for deformability indices for control, diabetic retinopathy and diabetic RBCs without diabetic retinopathy.

Figure 3 represents the scatter plot for all the cell cycles for Io and Imax using linear fit model in the control group. Figure 4 represents the scatter plot for all the cell cycles for Io and Imax using linear fit model for the study group for DM RBCs without DR. Figure 5 represents the scatter plot for all the cell cycles for Io and Imax using linear fit model for the study group for DR RBCs. Figure 6 represents the Kernel density plots for DI for both DR and control group. Figure 7 represents the Kernel density plots for Io and Imax for both DR and study groups.

**Figure 8:**
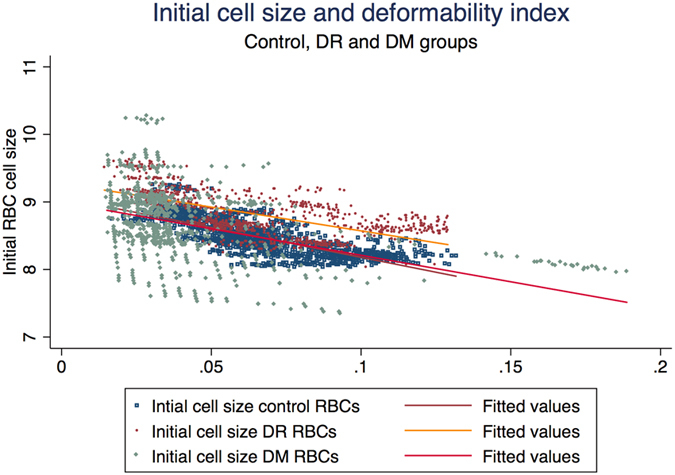
Scatter plot showing the difference between linear fit lines for control and study groups for deformability index and initial RBC cell sizes.

Using univariate regression analysis, we looked at biochemical factors in the blood and its correlation with DI. With DI as dependent variable, we did univariate regression analysis for all the biochemical and haematological parameters. None of the factors were found to be significantly correlated with DI on bivariate analysis. However DI was significantly correlated with initial cell size (Fig. 8). The scatter plot (Fig. 8) represents the negative correlation between DI and initial cell size (p = 0.02). The control RBCs had R^2^ of 0.608(p < 0.001) and DR RBCs had R^2^ of 0.4026 (p < 0.001) with DI of the respective RBCs. Hence further univariate analysis was done to determine the effect of haematological and biochemical parameters on the initial RBC cell size. As presented in Table 3, serum potassium, RBG, HbA1C, MCH had statistically significant influence on initial (basal) cell size. However, on multivariate analysis none of the factors seem to influence the initial cell size.

## Discussion

RBC deformability allows for normal blood flow in the microcirculation and also in the larger vessels at a high shear rate^9^,^10^. The deformability of RBC dictates the oxygen that reaches every part of the body through capillaries that are as small as approximately 3 μm^33^. The erythrocytes are enabled to do so by their geometry, the cytoplasmic viscosity due to the presence of haemoglobin (represented by MCHC) and viscoelasticity of the cell membrane^34^. It is widely believed and observed that all of these factors undergo a certain alteration in various pathophysiological conditions resulting in the RBC becoming more rigid and less deformable^9^,^10^,^11^,^13^,^18^,^34^,^35^,^36^,^37^,^38^,^39^,^40^. The ability of RBCs to carry out the process of deformation and retain their original shape at an individual level can be analysed in a broad spectrum of microcirculatory disorders paving way for effective molecular diagnostic tools. In our study, we investigated the deformability of RBCs and factors affecting the deformability in diabetic retinopathy which is the most common microvascular disorder affecting the eye.

There are various ways of studying the haemorheological characteristics of the blood dependent on the complexity of the disease model. Mathematical models and simulations to determine the mean shear modulus and bending modulus have been developed and tested before with a certain extent of endorsement^26^,^41^,^42^,^43^,^44^,^45^. The mechanistic behaviour of the outer membrane is researched to understand its stiffness with certain limitations in terms of explaining cytoplasmic viscosity and the inability of the cell to have a constant volume while undergoing deformability. A variety of mechanical tools are also employed to gain insight into the causes and effects of RBC deformability. The magnitude of forces applied, the corresponding displacement and the bandwidth at which these tools operate are the three main factors that allow us to choose the right set of techniques.

Optical tweezers use the momentum transfer from a strongly focused laser beam to trap objects such as cells, bacteria or even individual viruses^28^,^31^. Very often silica/polystyrene beads attached to the perimeter of the cell are used as convenient handles and may also increase the force that can be exerted on the cell^26^,^27^,^29^,^46^,^47^. For stretching experiments the cell under test is held by two laser beams (directly or attached to handles), and the separation between the beams increased to stretch the cell. The resulting cell elongation from this protocol is indicative of a cell’s deformability. Both elastic and viscoelastic properties through force versus displacement graphs can be studied. It is a relatively simple apparatus that can be made using an inverted microscope, coupled to a laser source. Image acquisition for data capture and analysis can be performed by a low cost CCD or CMOS camera^26^,^27^,^28^,^29^,^31^,^46^,^47^,^48^,^49^,^50^.

The forces applied on the cells are usually small, in the order of a hundred pico newtons. This is, however, sufficient to confine the cell against thermal fluctuations. By trapping the cell with two laser beams the orientation of the non-spherical RBC can also be controlled since the cell orients itself to maximise the volume within the foci. If one beam is stationary, while the other is mobile the cell can be stretched as the beam separation increases since the cell elongates to try to maintain the maximum overlap with the beams^29^,^31^. Dual beam optical tweezers can be used to study membrane fluctuation dynamics and its mechanical behaviour. The shear modulus, retrieved by image processing can be calculated using this technique^27^. In the current study, we used dual-beam optical tweezers to assess the deformability of the single cells, which not only allowed us to assess the deformability of several cells in the same blood sample but also enabled us to obtain baseline measurements of cell morphology such as initial cell size, surface area, volume of the cell and sphericity index of the cell.

Rheological properties of the RBCs are known to be sensitive to the altered microenvironment^37^. They can be affected by age, smoking, hypertension and other metabolic diseases. They are not only sensitive to the alterations in the metabolic condition of the tissue which is perfused, but also to the activities of the other co-existent cells which are in the immediate vicinity. Rheology of the RBCs can be altered secondary to the surrounding activated leukocytes or endothelium^37^,^51^. Incubation of the RBCs with activated leukocytes which produces oxygen free radicals can cause deleterious effects and these oxidative effects can in turn be inhibited by antioxidant enzymes. Biochemical alterations induced by oxidants include lipid peroxidation, haemoglobin oxidation, altered membrane proteins and lipid composition. This in turn affects RBC deformability and RBC adherence^52^. On the other hand, WBCs are known to directly affect the microvascular blood flow due to its increase rigidity but there are very limited reports on evaluating rigid characteristics of WBCs in diabetes^4^,^15^,^53^,^54^,^55^. Also total number of RBCs in circulation are much more in comparison to WBCs and it may well be postulated that both WBCs and RBCs have impact on microcirculation.

The results in our study were similar to previously published literature on RBC deformability in DM^15^,^32^,^53^,^55^. DI as computed by our proposed simple formula was found to be significantly affected in patients with DR. There was significant difference in initial cell size, final cell size, DI between DM RBC without DR and DR RBCs. The DR RBC was more swollen and less deformable as compare to DM RBC without DR. None of the haematological or biochemical factors in the blood were seen to be influencing the deformability of RBC in the current study which can be due to smaller patient sample size in our study. However, the larger initial cell size (Io) was significantly associated with reduced deformability. Also, the initial cell size of RBCs from patients with diabetes was significantly larger as compare to that of age matched control subjects. This implies that metabolic disturbances in diabetes can affect the cell size of the RBC which can eventually influence the deformability of the RBCs. MCH was associated with increased cell size on univariate analysis which suggests changes in intra-cytoplasmic viscosity leading to swollen RBCs. Also, on univariate analysis both HbA1C and blood glucose levels were associated with increased cell size suggesting association of impaired glycaemic control and osmotically swollen RBCs.

There are limited studies published on DI in DR and no studies have ever reported about the comparative size of the cell at the baseline in patients with T2DM. In the current study, we not only looked at the deformability index but also at the baseline size and morphology of single red blood cell in patients with T2DM in comparison to controls. This is the first study in the literature reporting about the deformability characteristics of RBCs in T2DM using dual beam optical tweezers though there are previously reported studies using cell transit analyser^56^. The latest version of cell transit analyser is able to measure deformability of more than 1000 cells at the same time but it has 2% total sampling error as there can be some haemolysed RBCs or small size RBCs filtered through the micropores and giving a falsely high and possibly erroneous deformable rate for RBCs^56^.

As we selectively identify the cells under the microscope prior to trapping , the total sampling error is almost negligible if at all any. In addition, we were also to identify initial cell size and final cell size for the cells and able to correlate the increase in cell size with DI using dual beam optical tweezers.

Diamtopoulos *et al*. reported about DI of RBC in DR in 1987^17^. The authors used filtration technique to assess DI in 69 patients with diabetes in comparison to 40 non diabetic healthy controls. They reported impaired DI in patients with DR which was directly proportional to severity of DR and postulated that it reflected diffuse metabolic disturbance^17^. Using microfluidic ektacytometer, Shin *et al*. demonstrated reduced elongation index (EI) in DM patients^57^. The authors demonstrated inverse correlation of EI with the levels of glycosylated haemoglobin and creatinine. Significant reduction in EI was noted in patients with combined diabetic nephropathy and retinopathy^57^. The microangiopathy changes correlated with changes in EI as concluded by the authors^57^. Keymel *et al*. used commercially available Laser-assisted optical rotational cell analyser (LORCA) to analyse EI in patients with DM and coronary artery disease (CAD). The authors reported significantly impaired RBC deformability in patients with DM and CAD and detrimental effect of plasma glucose concentration on RBC deformability^32^.

RDW was not found to be significantly associated with DI in our current study which can be due to relatively small sample size of our study. However, RDW provides the clinician information about the heterogeneity in shape and size of RBCs in blood and it has been shown to be associated with diabetes-associated complications^58^. In our study, even though we could identify significant changes in the cell size between control and study group and also that cell size adversely affected the DI, we could not establish any association with RDW, which is a measure of variation of volume of RBCs. Further research is warranted to identify association between RBC volume, RDW and impaired deformability.

Based on our results and previously published literature, we would like to postulate the following mechanisms (hypothesis) regarding impaired deformability of RBCs in T2DM^59^,^60^. Increased glycosylation, cholesterol and oxidative stress can result in altered cell morphology leading to formation of echinocytes or swollen RBCs with impaired deformability^60^. Also, increased blood glucose can result in glycosylation of haemoglobin, membrane and skeletal proteins^59^. There can be oxidative damage of the RBC membrane with oxidation of spectrin in the RBC cell membrane or reduced enzyme activities due to defective Na-K-ATPase pump^59^,^61^. In our study, there was increased potassium levels associated with increased cell size which possibly give us subtle clue about the altered membrane status in diabetic RBCs.

Under low shear conditions, blood behaves like non–Newtonian fluid resulting in apparently decrease viscosity with increase shear rate. With increasing shear rate, under low shear conditions (<10 s^−1^) viscosity decreases due to break down of RBC aggregates^62^. However, under high shear conditions (100 s^−1^), blood behaves like Newtonian fluid and no RBC aggregates are present. Blood deformability is predominantly determined by the haematocrit for Newtonian fluid but red cell deformability also has its own role to play in microcirculation; the more deformable the RBC , the less the propensity for increase in blood viscosity^63^. Our data indicates that there is probably implication of microvascular complications in DR and T2DM in association with reduced deformability of the RBCs and cell size.

## Conclusion

Dual-beam optical tweezers can be considered as a tool to investigate deformability of the single cells. We were able to demonstrate the effective application of this tool without the need for microbeads as handles for force application using dual-beam laser. Herewith, we have reported association of deformability with baseline cell size and have proposed a possible hypothesis about the inverse relationship. We have also demonstrated significantly swollen RBC in the T2DM in comparison to age matched healthy controls. The major limitation of this study is the relatively small sample size and future studies may allow us to establish the association between severity of diabetic retinopathy or diabetes associated complications and rheology of the blood.

## Additional Information

**How to cite this article**: Agrawal, R. *et al*. Assessment of red blood cell deformability in type 2 diabetes mellitus and diabetic retinopathy by dual optical tweezers stretching technique. *Sci. Rep.* **6**, 15873; doi: 10.1038/srep15873 (2016).

## Supplementary Material

Supplementary Information

Supplementary Video 1

## Figures and Tables

**Table 1:** Hematological and laboratory parameters for control and study (DR) group.

Hematological/Biochemicalvariables	Normal range(units)	**Controls (SD, 95% CI)**	**Patients (SD, 95%CI)**	pvalue(t test)
Hemoglobin (Hb)	13.0–17.0(g/dL)	14.27(1.64,12.90–15.64)	13.34(1.80, 11.67–15.01)	0.31
Hematocrit (Hct)	37–50%	42.12(0.04, 0.38–0.45)	39.47(0.04, 0.35–0.43)	0.27
Red blood cell count (RBC)	4.40–5.80(x10^12/L)	4.8(0.51, 4.37–5.22)	4.71(0.45, 4.51–5.01)	0.74
Mean corpuscular volume (MCV)	80–99	**87.81(2.81, 85.45**–**90.16)**	**83.48(3.22, 80.50**–**86.47)**	**0.01**
Mean corpuscular hemoglobin (MCH)	26.0–33.5(pg)	**29.68(1.04, 28.81**–**30.56)**	**27.84(1.82, 26.15**–**29.52)**	**0.02**
Mean corpuscular hemoglobin concentration (MCHC)	30.0–35.0(g/dL)	29.90(10,72, 20.94–38.87)	36.63(11.84, 32.53–34.72)	0.38
RBC distribution width (RDW)	11.5–15.0	13.58(1.48, 12.34–14.82)	13.65(1.37, 12.41–14.90)	0.92
Platelet count (PC)	150–400(x10^9/L)	270.5(76.63, 206.42–334.57)	317.57(69.54, 253.25–381.8)	0.23
Mean platelet volume (MPV)	7–13(fL)	10.73(1.41, 9.55–11.91)	10.88(0.77, 10.17–11.60)	0.81
White blood cell count (WBC)	3.0–10.0(x10^9/L)	7.07(1.63, 5.71–8.44)	7.79(1.25, 6.64–8.95)	0.36
Neutrophils	2.0–7.5(x10^9/L)	3.67(1.00, 2.83–4.51)	4.40(0.68, 3.76–5.03)	0.12
Lymphocytes	1.2–3.65(x10^9/L)	2.45(0.86, 1.73–3.18)	2.55(0.71, 1.89–3.21)	0.82
Monocytes	0.2–1.0(x10^9/L)	4.32(1.4, 3.11–5.53)	4.71(1.95, 2.90–6.52)	0.66
Eosinophils	0.0–0.4(x10^9/L)	3.5(0.9, 2.7–4.2)	3.14(0.2, 0.12–0.50)	0.66
Basophils	0.0–0.1(x10^9/L)	3.28(1.81, 1.77–4.80)	3.4(3.6, 0.67–6.73)	0.94
Erythrocyte sedimentation rate (ESR)	1–20(mm/hr)	7.87(6.26, 2.63–13.11)	21.14(28.81, –5.50–47.78)	0.22
Sodium (Na)	135–145(mmol/L)	142.62(2.26,140.73–144.51)	140(3.60, 136.66–143.33)	0.11
Potassium (K)	3.5–5.1(mmol/L)	4.46(0.36, 4.15–4.76)	4.77(0.18, 4.32–5.22)	0.18
Chloride (Cl)	98–107(mmol/L)	102.37(1.76, 100.89–103.85)	99.85(2.47, 97.56–102.14)	0.04
Bicarbonate	22–29(mmol/L)	23.62(2.50, 21.53–25.71)	24.14(2.11, 22.18–26.09)	0.67
Urea	1.7–8.3(mmol/L)	4.61(1.59, 3.28–5.94)	6.31(2.90, 3.62–8.99)	0.17
Creatinine	66–112(mmol/L)	83(24.85, 62.22–103.77)	92.71(22.42, 71.97–113.45)	0.44
Bilirubin	0–20(umol/L)	5.375(2.77, 3.05–7.69)	6(3.10, 3.12–8.87)	0.69
Alkaline phosphatase (ALP)	40–129(IU/L)	64(12.4,53.51–74.48)	89.71(18.10, 72.96–106.46)	<0.001
Aspartate aminotransferase (AST)	0–37(IU/L)	22.62(5.65, 17.89–27.35)	23.71(15.25, 9.61–37.81)	0.85
Alanine transaminase (ALT)	10–50(IU/L)	23.12(10.80, 14.09–32.15)	37.71(23.68, 15.81–59.61)	0.14
Protein	63–83(g/L)	**67.37(3.50, 64.44, 70.30)**	**73.28(3.77, 69.79**–**76.77)**	**0.007**
Albumin	34–50(g/L)	43.62(2.77, 41.30–45.94)	43.57(3.35, 40.46–46.67)	0.97
Globulin	19–35(g/L)	**23.75(2.54, 21.61**–**25.88)**	**29.71(1.88, 27.96**–**31.46)**	**<0.001**
C-reactive protein (CRP)	0–5.0(mg/L)	2.18(2.33, 0.23–4.14)	2.12(2.34, 0.04–4.29)	0.962
Random blood glucose (RBG)	3.9–6.9(mmol/L)	**4.91(0.40, 4.58**–**5.24)**	**10.04(3.67, 6.64**–**13.43)**	**0.001**
Glycosylated hemoglobin (HbA1C)	4.5–6.0%	**5.82(0.34, 5.54**–**6.11)**	**9.77(2.64, 6.17**–**9.15)**	**0.001**
Triglycerides (TG)	0.0–2.2(mmol/L)	1.62(0.98, 0.80–2.44)	2.31(2.10, 0.36–4.26)	0.42
Cholesterol	2.3–4.9(mmol/L)	4.93(1.18, 3.94–5.93)	4.47(1.18, 3.94–5.93)	0.38
High density lipoprotein (HDL)	0.9–1.5(mmol/L)	1.34(0.31, 1.05–1.63)	1.17(0.29, 1.08–1.42)	0.29
Low density lipoprotein (LDL)	0–3.0(mmol/L)	2.82(0.84, 2.12–3.52)	2.76(0.72, 2.00–3.53)	0.89
Angiotensin converting enzyme (ACE)	8–52 Units/liter	35.87(11.63, 26.15–45.60)	31(26.41, 6.56–55.43)	0.64
Fibrinogen	2–4 (g/L)	3(0.59, 2.50–3.5)	3.12(0.89, 2.29–3.95)	0.74

**Table 2:** RBC deformability indices using dual-beam optical tweezers for healthy control subjects and study subjects (diabetic retinopathy).

	**Control group**	**DM group**	**DR group**	
Total number of cell cycles (5–10cycles/cell)	925	925	925	
Average unstretched cell size (μm) (SD, Range)	8.45(±0.25, 8.03–9.25)	8.68(±0.49, 7.33–10.27)	8.82(±0.32, 8.03–9.62)	p < 0.001
Avergae maximal stretched cell size (μm) (SD, Range)	9.04(±0.17, 8.55–9.65)	9.23(±0.49, 7.82–10.56)	9.39(±0.26,8.70–10.03)	p < 0.001
Average difference between stretched and unstretched cell size (μm) (SD, 95CI)	0.59(±0.19, 0.57–0.60)	0.56(±0.32, 0.49–0.51)	0.56(±0.24, 0.55–0.58)	P = 0.009
Deformability Index (SD, 95CI)	0.0698(±0.024, 0.068–0.072)	0.0645(±0.03, 0.063–0.067)	0.0635(±0.029, 0.062–0.066)	p < 0.001

SD- Standard deviation, DM- Diabetes mellitus, DR – Diabetic retinopathy

**Table 3:** Univariate regression analysis affecting the initial cell size:-

	**R-squared**	**95% CI**	**P-value**
Potassium	0.334	0.047–0.565	0.024
Random blood glucose	0.424	0.012–0.071	0.008
HbA1C	0.406	0.019–0.096	0.006
MCH	0.274	−0.14 to–0.001	0.045

MCH – Mean corpuscle hemoglobin, HbA1C – glyosylated hemoglobin
