# Supplementary material for: Assessment of red blood cell deformability in type 2 diabetes mellitus and diabetic retinopathy by dual optical tweezers stretching technique
Source: Sci Rep. 2016 Mar 15;6:15873. doi: 10.1038/srep15873 (PMC4792142; doi:10.1038/srep15873)
Supplement: Supplementary Information [file srep15873-s1.doc]

**Assessment of red blood cell deformability in type 2 diabetes mellitus and diabetic retinopathy by dual optical tweezers stretching technique**

Dr Rupesh Agrawal,^1,2,3,4^ Mr Thomas Smart,^5^ Dr João Cardoso,^1^ Mr Christopher Richards,^5^ Ms Rhythm Bhatnagar,^2^ Mr Adnan Tufail,^1^ Prof David Shima,^3^ Dr Phil Jones,^5^ Mr Carlos Pavesio^1,3^

**^1^**Moorfields Eye Hospital NHS Foundation Trust, London, UK.^2^ Department of Mechanical Engineering, University College London, London, UK. ^3^Institute of Ophthalmology, University College London, London, UK. ^4^National Healthcare Group Eye Institute, Tan Tock Seng Hospital, Singapore ^5^Department of Physics and Astronomy, University College London, London, UK.

**Supplemental Information:**

**Slide Preparation**

**Material Requirements**: Well Slides, Cover slip, Pipettes, Kim Wipes, 1ml eppendorfs, Microscope Oil, Nail Paint, PBS (Phosphate Buffered Saline) Solution, BSA (Bovine Serum Albumin), Ethanol, Methanol

**Procedure**

1. Clean the slide with ethanol and PBS and wipe it properly with Kim Wipes till it’s dry. Keep the slide aside on a clean surface, preferably a sheet of wipes.
2. Take a 1mL eppendorf and rinse it with PBS twice to clean any dirt deposition. Fill the eppendorf with PBS till the 1mL mark using the 20-200 ul pipettes.
3. Pipette 1 uL of whole blood from the EDTA bulb and mix it with the PBS in the eppendorf. Cap the eppendorf and roll it gently to allow mixing of blood. Place the EDTA bulb back in the bio-box in case for later usage.
4. Clean the spatula with ethanol and dry it completely with the wipes. Take about 1 mg of BSA and add it to the solution and mix it gently.
5. Clean the cover slip with ethanol and PBS and wipe it dry.
6. Pipette 100-150μl of the solution (PBS+BSA+Blood) on the slide. Gently slide the coverslip from an angle to spread the wet mount. Dry off excess sample properly with Kim Wipes.
7. Use the oil dropper to put oil over the slip and seal with nail paint from all sides. Label the slide with the marker (Sample Code and Date) to prevent mixing of multiple slides if dealing with 2 specimens.

**Precautions/Additional Notes**

1. Always ensure that the ethanol dries off completely to prevent any mixing with the blood.
2. Store BSA at 4°C.
3. Ensure the blood/solution come as little as possible in contact with the air to prevent any contamination (often echinocytes).
4. Clean the slide with ethanol and methanol after you’re done and keep the water running over the slide and in the basin since methanol fumes are toxic. Repeat the same procedure for the spatula. Place them on the net to dry.
5. In case the concentration of RBCs is large, dilute the solution with PBS till a light pink/almost transparent solution is obtained.
